# Cannabinoid CB1 Receptor Expression and Localization in the Dorsal Horn of Male and Female Rat and Human Spinal Cord

**DOI:** 10.1080/24740527.2023.2264895

**Published:** 2023-11-28

**Authors:** Jessica Parnell, Newton Martin, Annemarie Dedek, Christopher Rudyk, Jeffrey Landrigan, Justin Bellavance, Simon VanDerLoo, Eve C. Tsai, Michael E. Hildebrand

**Affiliations:** a Department of Neuroscience, Carleton University, Ottawa, Ontario, Canada; b Neuroscience Program, Ottawa Hospital Research Institute, Ottawa, Ontario, Canada; c Brain and Mind Research Institute, University of Ottawa, Ottawa, Ontario, Canada; d Division of Neurosurgery, Department of Surgery, The Ottawa Hospital, Ottawa, Ontario, Canada

**Keywords:** pain, spinal cord, dorsal horn, cannabinoid, CB1, CB2, sex, human

## Abstract

**Background:**

Preclinical and clinical evidence suggests that cannabis has potential analgesic properties. However, cannabinoid receptor expression and localization within spinal cord pain processing circuits remain to be characterized across sex and species.

**Aims:**

We aimed to investigate the differential expression of the cannabinoid type 1 (CB1) receptor across dorsal horn laminae and cell populations in male and female adult rats and humans.

**Methods:**

To investigate and quantify CB1 receptor expression in the spinal dorsal horn across species, we refined immunohistochemical procedures for successful rat and human fixed tissue staining and confocal imaging. Immunohistochemical results were complemented with analysis of CB1 gene (*CNR1*) expression within rodent and human dorsal horn using single-cell/nuclei RNA sequencing data sets.

**Results:**

We found that CB1 was preferentially localized to the neuropil within the superficial dorsal horn of both rats and humans, with CB1 somatic staining across dorsal horn laminae. CB1 receptor immunoreactivity was significantly higher in the superficial dorsal horn compared to the deeper dorsal horn laminae for both rats and humans, which was conserved across sex. Interestingly, we found that CB1 immunoreactivity was not primarily localized to peptidergic afferents in rats and humans and that *CNR1* (CB1) but not *CNR2* (CB2) was robustly expressed in dorsal horn neuron subpopulations of both rodents and humans.

**Conclusions:**

The conserved preferential expression of CB1 receptors in the superficial dorsal horn in male and female rodents and humans has significant implications for understanding the roles of this cannabinoid receptor in spinal mechanisms of nociception and analgesia.

## RÉSUMÉ

**Contexte:** Les données probantes précliniques et cliniques indiquent que le cannabis possède des propriétés analgésiques potentielles. Cependant, l’expression et la localisation des récepteurs cannabinoïdes au sein des circuits de traitement de la douleur de la moelle épinière restent à caractériser selon le sexe et les espèces.

**Objectifs:** Nous avons cherché à étudier l’expression différenciée du récepteur cannabinoïde de type 1 (CB1) dans les différentes couches de la corne dorsale et les populations cellulaires chez des rats et des êtres humains adultes de sexe masculin et féminin.

**Méthodes:** Pour étudier et quantifier l’expression des récepteurs CB1 dans la corne dorsale de la moelle épinière chez différentes espèces, nous avons perfectionné les procédures d’immunohistochimie pour obtenir des résultats de coloration réussis sur des échantillons de tissus provenant de rats et d’êtres humains, ainsi que des images confocales. Les résultats immunohistochimiques ont été complétés par l’analyse de l’expression du gène CB1 (CNR1) dans la corne dorsale des rongeurs et des humains en utilisant des ensembles de données de séquençage d’ARN au niveau des cellules uniques et des noyaux.

**Résultats:** Nous avons constaté que le CB1 était principalement localisé dans le neuropile au sein de la corne dorsale superficielle chez les rats et les humains, avec une coloration somatique du CB1 dans les différentes couches de la corne dorsale. Chez les deux espèces, l’immunoréactivité du récepteur CB1 était significativement plus élevée dans la couche superficielle de la corne dorsale par rapport aux couches plus profondes, indépendamment du sexe. De manière intéressante, nous avons constaté que l’immunoréactivité du CB1 n’était pas principalement localisée dans les afférences peptidergiques chez les rats et les humains. De plus, nous avons observé une forte expression du gène CNR1 (CB1), mais pas du CNR2 (CB2), au sein de sous-populations de neurones de la corne dorsale chez les rongeurs et les êtres humains.

**Conclusions:** La localisation privilégiée et constante des récepteurs CB1 dans la couche superficielle de la corne dorsale chez les rongeurs et les humains, quel que soit leur sexe, revêt une importance majeure pour la compréhension des fonctions de ce récepteur des cannabinoïdes dans les mécanismes médullaires de la nociception et de l’analgésie.

## Introduction

Chronic pain represents a debilitating health crisis that has been intensified by the lack of pharmaceutical treatments that are both safe and effective. In terms of traditional medicines, cannabis contains natural compounds that have been used for medical purposes, including to treat pain, for millennia.^1^,^2^ Over the last few decades, a combination of preclinical rodent and clinical human studies have led to the proposal that cannabis and cannabis-based medicines have direct mild to moderate analgesic effects while producing minimal adverse side effects.^3–5^ However promising these emerging results may be, the International Association for the Study of Pain Presidential Task Force on Cannabis and Cannabinoid Analgesia recently concluded that they could not endorse the general use of cannabis for pain relief because of significant research gaps.^6^ These gaps include testing whether the molecular determinants of cannabinoid-mediated pain regulation are conserved or diverge across key translational variables such as sex and species.^7,8^

The superficial dorsal horn (SDH) of the spinal cord is a critical hub for nociceptive processing and is implicated in cannabinoid-driven analgesia. Excitatory afferents from peripheral nociceptive dorsal root ganglia (DRG) neurons terminate primarily in laminae I and II of the SDH, with a small subset of projection neurons in lamina I transmitting integrated nociceptive signals onto the cortical areas of the brain pain matrix.^9^ In contrast, neurons in the deeper dorsal horn (DDH), including laminae III–VI, primarily process other somatosensory modalities as well as premotor spinal functions.^10^ Intrathecal (spinal) injections of exogenous cannabinoids produce antinociceptive effects in mice,^11^ and intrathecal blockers of a specific subtype of cannabinoid receptors, cannabinoid type 1 (CB1) receptors, increase pain responses.^12^ These data suggest an important role of the spinal endocannabinoid system, and particularly CB1, in the homeostatic modulation of pain.^13,14^

The endocannabinoid system regulates excitability within the peripheral and central nervous systems and consists of endogenous cannabinoid compounds (endocannabinoids), cannabinoid receptors, and endocannabinoid synthesis and degradation enzymes.^13,14^ Endocannabinoids regulate neuronal excitability through the activation of G protein–coupled cannabinoid receptor types 1 and 2 (CB1 and CB2, respectively). CB1 receptors are preferentially localized to central presynaptic terminals, whereas CB2 receptors are found mainly in nonneuronal cells in the periphery. Within the peripheral–spinal nociceptive circuits of male rodents, CB1 receptors are highly expressed in subpopulations of DRG neurons and localized to presynaptic primary afferents but are also expressed in a subset of postsynaptic dorsal horn neurons.^15–18^ Though CB2 receptors are not expressed in DRG or spinal cord neurons of naïve male rodents, their expression can be induced following nerve injury.^19^ At central synapses, the activation of CB1 receptors drives a sustained reduction in neurotransmitter release and resultant endocannabinoid-mediated long-term depression.^20^ Within spinal circuits, administration of CB1 agonists inhibits the Aδ- and C-fiber-mediated activation of dorsal horn neurons *ex vivo*^21^ and selectively attenuates noxious stimuli-evoked activity of dorsal horn neurons *in* *vivo* in male rats.^22^

As exemplified above, evidence on cannabinoid receptor localization and function in dorsal horn pain processing circuits has mainly been restricted to male rodents. However, dramatic sex differences in spinal mechanisms of nociception are now emerging,^8,23^ and the administration of cannabinoids or cannabinoid receptor agonists produces a stronger analgesic response in female compared to male rats at baseline.^24–26^ Several clinical studies have revealed that, similar to rodents, women tend to have increased sensitivity to the psychological and physiological effects of cannabis compared to men.^27^ For CB1 receptors specifically, various groups have identified sex- and region-specific differences in CB1 expression within rodent brains,^28–31^ but analogous studies have not been completed for rodent spinal cord. Moreover, there is no literature characterizing baseline CB1 expression across sex for human nociceptive regions, including the dorsal horn. We therefore leveraged our ability to collect viable spinal cord tissue from human organ donors^23,32^ to systematically compare CB1 expression across dorsal horn nociceptive and non-nociceptive regions in male versus female rats and humans.

## Materials and Methods

### Animals

Spinal cord tissue was used from male and female adult Sprague-Dawley rats (3–4 months old) purchased from Charles River. Animals were given food and water *ad libitum* and were kept on a 12-h light cycle (lights on 7 am–7 pm). Rats were paired housed in cages with corn cob bedding with Enviro-dry, a PVC tube, and a nylon chew for enrichment. Rats were housed for 7 to 14 days before experiments began and were euthanized between 11 am and 3 pm. Experimental procedures using these animals were approved by the Carleton University Animal Care Committee (Protocol# 117623) and performed in accordance with the guidelines set by the Canadian Council for Animal Care.

### Rat Spinal Cord Isolation and Preparation

Twelve adult Sprague-Dawley rats (six females and six males) were used for this study. First, the rats were anesthetized with an intraperitoneal injection of 3 g/kg urethane. Next, the spinal cord was isolated and removed via posterior laminectomy; then the meninges were removed and the nerve roots trimmed. The cord was then immediately placed in ice-cold protective sucrose solution (50 mM sucrose, 92 mM NaCl, 17 mM D-glucose, 26 nM NaHCO_3_, 5 mM KCl, 1.25 mM NaH_2_PO_4_, 0.5 mM CaCl_2_, 7 mM MgSO_4_), bubbled with carbogen (95% O_2_, 5% CO_2_) to preserve the tissue prior to fixation. The tissue was then fixed in a 4% PFA: paraformaldehyde solution in PB: phosphate buffer for 24 h at 4°C, washed in 10% sucrose solution in PB for 24 h, washed again in 10% sucrose solution in PB for 6 to 24 h, and then finally placed in 30% sucrose solution in PB for at least 72 h, all at 4°C.

For spinal cord freezing, tissue sections were embedded in cryomatrix and frozen in isopentane that was chilled with liquid nitrogen and then stored at −80°C before sectioning. The rat spinal cord tissue was sectioned at 25 *μ*m on a microtome cryostat at −20°C and mounted immediately onto microscope slides in serial fashion and stored at −80°C in preparation for immunohistochemical experiments.

### Human Donors and Tissue Collection

Adult (20–70 years old, rounded to the nearest 5 for privacy reasons) male and female human spinal cord tissue was collected from organ donors identified by the Trillium Gift of Life Network, as previously described.^23,32,33^ Informed written consent was obtained from the donor’s family prior to collection. Ethics approval was obtained to collect and conduct experiments with human tissue by the Ottawa Health Science Network Research Ethics Board (Protocol ID No. 20150544–01 H) and the Carleton University Research Ethics Board B (Ethics Protocol No. 104836). Donors were prescreened to exclude any blood-borne illnesses such as human immunodeficiency virus, hepatitis, and syphilis. Donors with chronic illnesses such as cancer and chronic pain or with damage to the spinal cord were excluded from the study to prevent any confounding variables from interfering with our results.

During the human tissue collection, a cooling bed was used to induce hypothermia and the body was perfused with a preservation solution designed to prolong the viability of the organs while without blood and oxygen (e.g., Custodiol HTK Solution or Perfadex Plus). Once the organs were removed for transplant, the vertebral column was opened to isolate and remove the spinal cord within 1 to 3 h of aortic cross-clamping or flushing the body with protective solutions. The dura mater was removed, and thoracic or lumbar sections of the spinal cord were cut into 6- to 10-mm sections and placed in ice-cold sucrose cutting solution (50 mM sucrose, 92 mM NaCl, 15 mM glucose, 26 mM NaHCO_3_, 5 mM KCl, 1.25 mM NaH_2_PO_4_, 0.5 mM CaCl_2_, and 7 mM MgSO_4_) to preserve the tissue in preparation for immunohistochemical experiments. The spinal cord was then fixed in a 4% PFA solution in PB for 24 to 36 h at 4°C, washed in 10% sucrose solution in PB for 24 h, rewashed in 10% sucrose solution in PB for 6 to 24 h, and then finally placed in 30% sucrose solution in PB for at a minimum of 72 h, all at 4°C.

For spinal cord tissue freezing, the fixed human tissue sections were embedded in cryomatrix and then frozen in isopentane that was chilled with liquid nitrogen and stored at −80°C prior to sectioning. The human spinal cord tissue was sectioned at 25 to 35 *μ*m on a microtome cryostat at −20°C and immediately mounted onto microscope slides in serial fashion and stored at −80°C in preparation for immunohistochemical experiments.

### Free-Floating Tissue Preparation

A subset of human spinal cord tissue was prepared as free-floating slices, as opposed to slide-mounted. For these samples, the extraction technique in the operating room was identical to the procedure described above; however, instead of immediately fixing the tissue in 4% PFA, the tissue was bubbled in saline for 70 min before being fixed in 4% PFA for 24 to 36 h (because this tissue was also used as a control for experiments as described in Dedek et al.^32^). The tissue was then washed in 10% sucrose solution in PB for 24 h, washed for a second time in 10% sucrose solution for 6 to 24 h, and finally placed in 30% sucrose solution for 72 h. The tissue sections were then placed in cryoprotectant solution (28.7 mL of sodium phosphate monobasic dihydrate 31.2 g/L [pH 7.3], 96.3 mL of sodium phosphate dibasic anhydrous 28.4 g/L [pH 7.3], 375 mL of diethylpyrocarbonate water, 300 mL of ethylene glycol, and 200 mL of glycerol) at −20°C until sectioning. Transverse sections of the spinal cord were sliced at 25 *μ*m using a Leica SM2000R microtome and stored again in cryoprotectant solution at −20°C in preparation for immunohistochemical experiments.

### Immunohistochemistry

Immunohistochemical methods were used to investigate the distribution pattern of CB1 receptors in the spinal dorsal horn of adult rats and humans. After 3 × 5 min washes in PBS, the tissue was incubated in a peroxidase blocking solution (50% methanol, 48.2% PBS, and 1.8% hydrogen peroxide) for 30 min at room temperature. Following the peroxidase block, the tissue was washed 3 × 5 min in PBS and blocked for 1 h in a PBS solution containing 5% NGS: normal goat serum, 0.3% 10 M Triton-X, and 0.3% BSA: bovine serum albumin. The blocker was then pipetted out and the tissue was incubated in a solution containing the primary antibodies against the CB1 receptor: rabbit anti-CB1 (1:1000, Immunogenes) and antibodies against calcitonin gene–related peptide (CGRP): mouse anti-CGRP (1:5000), diluted in the blocking solution (described above) for 48 h at 4°C. This CB1-targeting primary antibody from Immunogenes has been previously optimized for CB1 staining and validated using CB1 knockout mice.^34^ Following incubation in primary antibody, the tissue sections were washed 3 × 10 min with PBS and incubated with secondary antibodies: goat anti-mouse AlexaFluor 647 (1:1000) and goat anti-rabbit immunoglobulin G conjugated with horseradish peroxidase (1:500) for 2 h at room temperature and protected from light. Next, the slides were washed 3 × 10 min PBS and incubated for 10 min at room temperature in tyramide signal amplification (TSA) diluted 1:50 in amplification diluent (TSA plus Cyanine 3 System) to amplify the CB1 signal. The slides were washed 3 × 10 min in PBS and incubated in Hoechst 33,258 (1:1000) diluted in PBS for 5 min to stain for nuclei. The tissue was then washed for one last round of 3 × 10 min wash in PBS before being mounted with Fluoromount and covered with a coverslip. The slides were sealed with clear nail polish within 24 to 48 h of placing the coverslip to prevent the tissue from drying out.

### Image Acquisition

All images were acquired using a Zeiss LSM 800 AxioObserverZ1 confocal microscope and further processed using Zen 2.6 software. Laser intensity was appropriately chosen to limit photobleaching of the sample, and identical microscope settings were used for all images to maintain continuity. Tiling was used to obtain an image of the entire dorsal horn, and appropriate z-stacks were obtained to create a hyperstack to account for multiple layers of tissue. The tiled images were stitched in Zen 2.6 software and then further analyzed in ImageJ.

### Image Analysis

**Figure 1.**
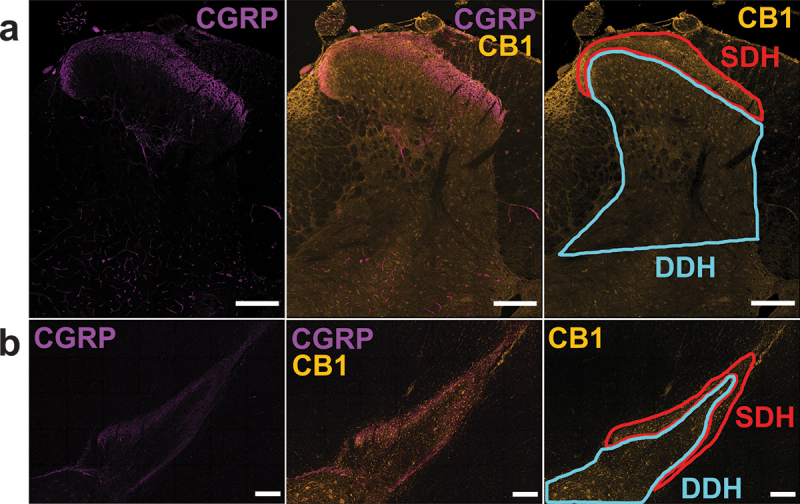
Immunoreactivity of CB1 receptors in the CGRP-positive SDH region and CGRP-negative DDH region. Representative immunopositive images from a (A) lumbar female rat and (B) thoracic male human dorsal horn showing prominent staining for CGRP in laminae I and IIo of the SDH (left, purple), overlaid with CB1 immunolabelling (middle, yellow). Right: Immunoreactivity for the CB1 receptor including the analysis outline for the SDH based on the leftmost CGRP staining (red outline) and the blue analysis outline that represents the remaining DDH region. Scale bar = 200 µm.

Quantification of CB1 receptor expression was obtained and analyzed using ImageJ. Two main regions are of interest in this study: the SDH and the DDH. Using CGRP as a marker for the SDH, as previously done by Eftekhari and Edvinsson,^35^ a contour line was drawn to outline the SDH where there was maximal CGRP-positive staining in both rat (Figure 1a) and human (Figure 1b) spinal sections. Using the contour tool, a second selection was made to encompass the DDH.

We used ImageJ to measure the optical density (O.D.) of the cy3-stained areas (representing CB1 receptors) in the SDH and then the DDH, which was divided by the area of each respective region. All values were normalized to O.D./area values from a background region, which was determined by a square selection of the white matter in the dorsal column, medial to the dorsal horn, where there should be minimal cy3 fluorescence. Comparisons in receptor expression were made between the SDH and DDH regions in each animal and human and then further compared between sex and species.

### Mouse and Human Single-Cell RNA Sequencing Data Information and Analysis

Fully clustered mouse and human single-cell/nuclei RNA sequencing data were shared with us by Dr. Ariel Levine and Dr. Kaya Matson from the National Institute of Neurological Disorders and Stroke in Maryland.

The mouse single-cell RNA sequencing data originated from a meta-analysis of six individual studies that performed single-cell/nuclei RNA sequencing on mouse lumbar spinal cord tissue.^36^ Neurons were clustered into excitatory or inhibitory subtypes such as Excit-1 (E1) or Inhib-2 (I2) using a combination of statistical and computational techniques as well as a comparison with well-established cell types in scientific literature. Using the laminar location of each neuronal cluster identified through in situ hybridization by Russ and colleagues,^36^ we further compiled these subtypes into SDH or DDH groupings whereby the SDH category included the clusters from laminae I, II, and/or IIo and the DDH category included clusters from laminae II, IIi, and/or III–VI.

The human spinal cord single-cell RNA sequencing data originated from a study that performed single-nucleus RNA sequencing on highly viable human donor lumbar spinal cord tissue from four male and three female donors aged approximately 20 to 80 years (ages rounded for privacy).^37^ The neuronal cells were clustered into excitatory or inhibitory neuronal subtypes based on the high expression of established excitatory or inhibitory genes such as SLC17A6 for excitatory and GAD1, GAD2, PAX2, and SLC6A5 for inhibitory subpopulations. Yadav and colleagues found that most of the neurons in the dorsal group originated from the SDH (laminae I–II), with a few exceptions.^37^

GScpter is a modular low-code pipeline developed by Newton Martin, Justin Bellavance, and Simon Vanderloo to investigate target-specific single-cell RNA sequencing gene expression across user-defined cluster groups.^38^ It was used for all of the single-cell RNA sequencing data presented here. In this study, average expression was calculated using the average counts per million (CPM) by dividing each count by the total number of counts, multiplying by 1 million, and then taking the average CPM across all cells in a group. Percent expressed in GScpter was calculated for each gene by dividing the number of cells that have greater than 0 RNA counts by the total number of cells in the group and then multiplying by 100. Neurons were grouped into the original cluster groupings from Russ et al.^36^ and Yadav et al.^37^ and were subsequently grouped further across SDH or DDH and across DH overall. The average expression and percent expressed of *CNR1* and *CNR2* were displayed using dot plots. The percent expressed was represented by the size of each dot. The average expression was represented by the color of the dot. To map the average expression on a color scale, the log_10_ of one plus the average CPM was placed on a *z*-score as previously reported by Yadav and colleagues.^37^

### Statistical Analysis

All statistical tests were completed using SPSS Statistics software. Data are presented as mean ± standard error of the mean. For all rodent data, each animal was immunostained in triplicate and a mean of the three slides was obtained to create a mean for each animal. For all human data, each donor was immunostained in duplicate and a mean of the two slides was obtained to create a mean for each human. Levene’s test was used to test for homogeneity of variance, and the Shapiro-Wilk test was used to test for normality. When assumptions for parametric tests were met, a one-way analysis of variance (ANOVA) was used (unpaired samples). When assumptions were violated, in the case of paired samples, the Wilcoxon signed-rank test was used, and the Mann-Whitney *U* test was used for independent samples. A two-way aligned rank transform ANOVA^39,40^ was used to compare groups for two-factor analysis. For all tests, *P* < .05 was considered statistically significant. For detailed results of statistical tests, please see Supplementary Table S1.

## Results

### CB1 Localization within the Dorsal Horn Is Conserved between Male and Female Rats

**Figure 2.**
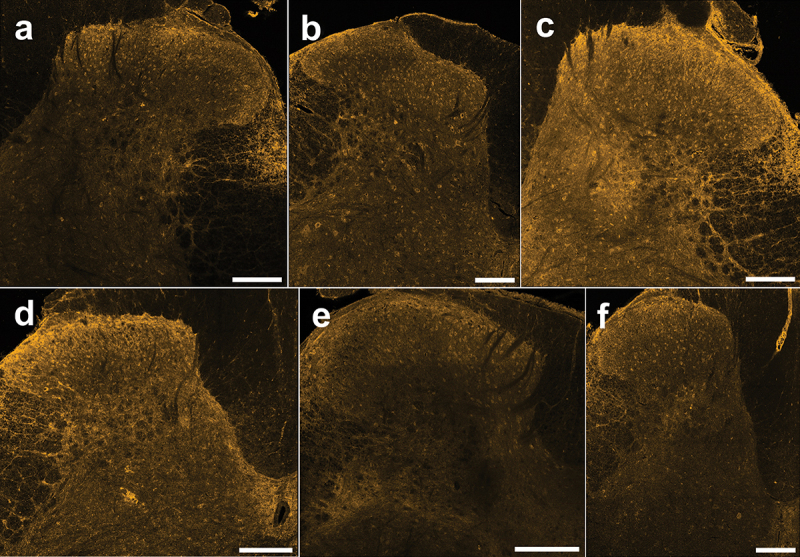
Immunoreactivity of the CB1 receptor in lumbar spinal cord sections from female rats. (A)–(F) Representative confocal images (20× objective) of CB1 immunostaining from each female rat (*n* = 6) showing neuropil staining that is especially pronounced in the SDH and cellular staining across the DH. Scale bar = 200 µm.

**Figure 3.**
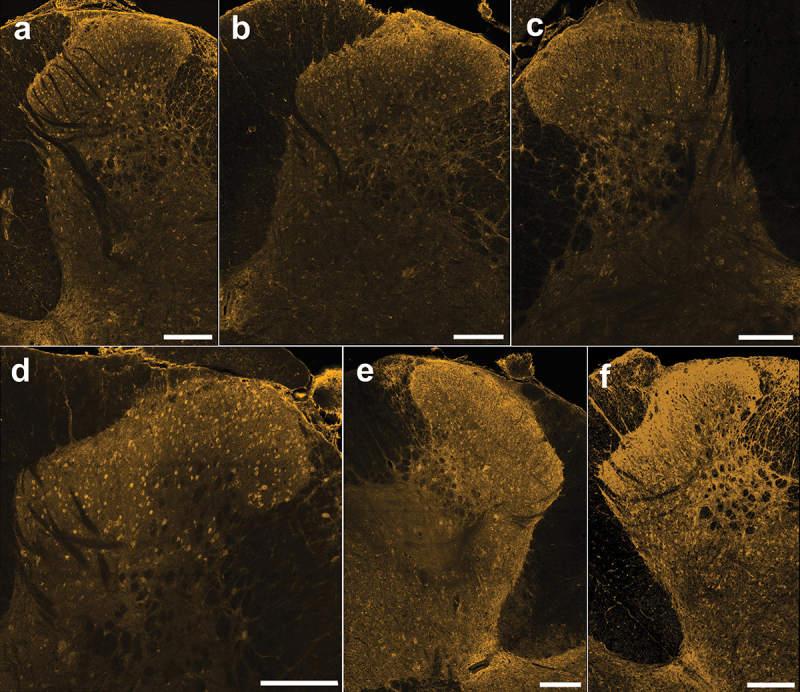
Immunoreactivity of the CB1 receptor in lumbar spinal cord sections from male rats. (A)–(F) Representative confocal images (20× objective) of CB1 immunostaining from each male rat (*n* = 6) showing neuropil staining that is especially pronounced in the SDH and cellular staining across the DH. Scale bar = 200 µm.

To investigate the baseline expression of cannabinoid CB1 receptors in pain processing regions of the spinal cord, we immunostained adult rat spinal cord sections using a knockout-validated CB1 antibody combined with TSA for optimized receptor labeling. We identified the relatively pain-specific SDH region of the dorsal horn through co-staining against a neuropeptide that is released from peptidergic C-fiber afferents in laminae I and II (CGRP^41,42^; Figure 1a). Qualitatively, we found that CB1 immunoreactivity was localized to a combination of punctate neuropil as well as somatic labeling across the rat dorsal horn, with stronger immunoreactivity within the SDH (Figures 2 and 3). Unlike that found for N-methyl-D-aspartate receptors,^43^ we observed that CB1 staining intensity was consistent across the mediolateral axis of the SDH in male and female adult rats. We observed the same pattern of CB1 dorsal horn staining between the lumbar (Figures 2 and 3) and thoracic (Figure S1) rat spinal cord. Control experiments on rat and human tissue with no primary antibody confirmed that this CB1 staining pattern was not due to secondary antibody background staining or tissue autofluorescence (Figure S2). Thus, we conclude that CB1 receptors are localized to both neuropil and cell bodies within the dorsal horn of both female and male adult rat spinal cord.

### CB1 Is Preferentially Localized to the SDH in Male and Female Adult Rats

**Figure 4.**
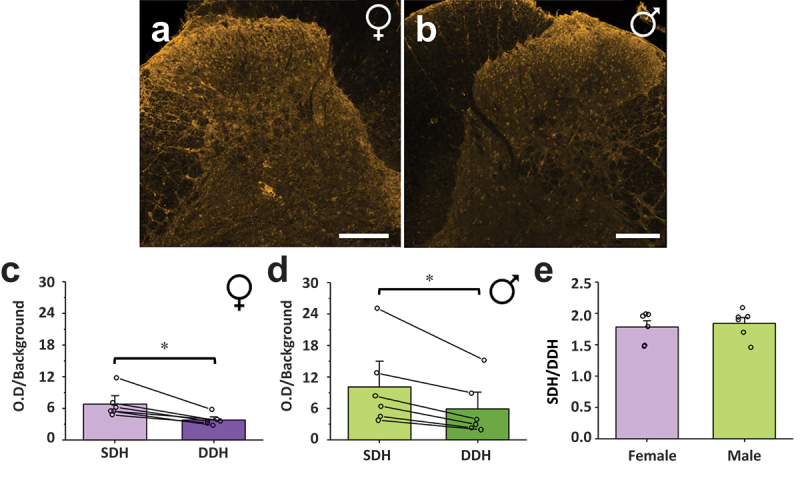
CB1 receptor immunoreactivity is increased in the SDH compared to the DDH in female and male rats. Representative confocal images of CB1 immunoreactivity in the dorsal horn of (A) female and (B) male rats, side by side for direct comparison. The normalized CB1 optical density in the SDH of rats is statistically higher than the normalized optical density in the DDH for (C) female and (D) male rats. *P < .05. (E) A ratio comparing the normalized CB1 optical density of the SDH versus the DDH reveals that the increased optical density of CB1 receptors in the SDH of rats is conserved across sex. *n* = 6. Scale bar = 200 µm.

We next aimed to quantify and statistically compare the relative localization of CB1 receptors to the pain processing SDH versus the somatosensory/premotor processing DDH region in male and female rats using an unbiased analysis approach. To achieve this, we selected the CGRP-positive region representing the SDH^35^ and the CGRP-negative region of the dorsal horn representing the DDH and measured the O.D. of CB1 immunoreactivity in each region, corrected to background (see Figure 1 for method). CB1 staining, imaging, and O.D. quantification were done in triplicate spinal sections for each experimental animal, with average values taken from these technical replicates for each region or ratio of interest. Using this approach, we found that CB1 immunoreactivity (O.D./background) in the SDH was significantly higher than in the DDH for both adult female (*n* = 6, *P* = .028, Figure 4a,c) and male (*n* = 6, *P* = .028, Figure 4b,d) rats (further statistical values are provided in Supplementary Table S1). To directly compare the extent of enhanced SDH localization across sex, we took the normalized CB1 O.D. in the SDH and divided it by the normalized O.D. in the DDH to create an SDH/DDH CB1 localization ratio (Figure 4e). In female rats, the average SDH/DDH ratio was 1.78 ± 0.10 (*n* = 6), which was not significantly (*P* = .69) different from the SDH/DDH ratio of male rats (1.84 ± 0.09, *n* = 6). Together, these results suggest that CB1 receptors are preferentially localized to the SDH in rats and that this CB1 localization is conserved across sex.

### Preferential Localization of CB1 Receptors to the SDH within the Human Spinal Cord

**Figure 5.**
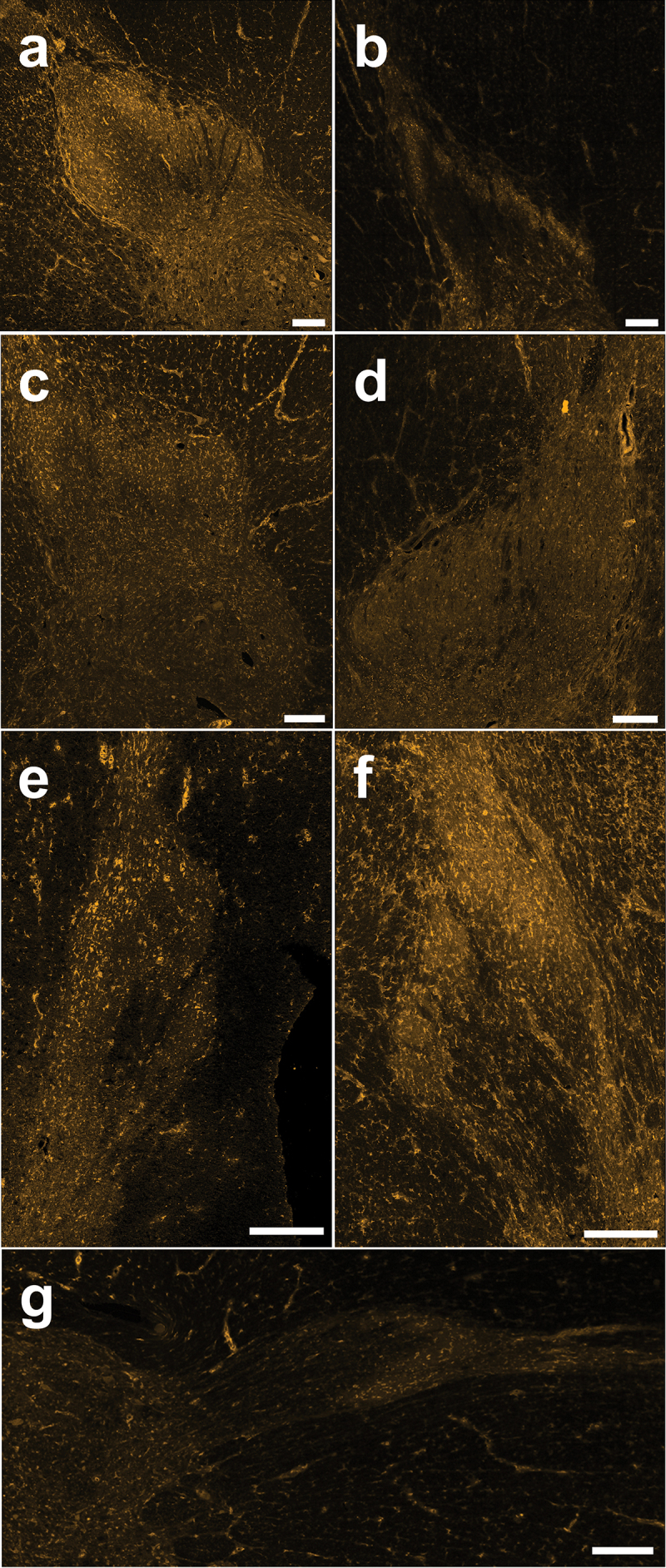
Immunoreactivity of the CB1 receptor in thoracic and lumbar spinal cord sections from female human organ donors. (A)–(G) Representative confocal images (20× objective) of CB1 immunostaining from each female human (*n* = 7) showing neuropil staining that is especially pronounced in the SDH and cellular staining across the DH. Scale bar = 200 µm.

**Figure 6.**
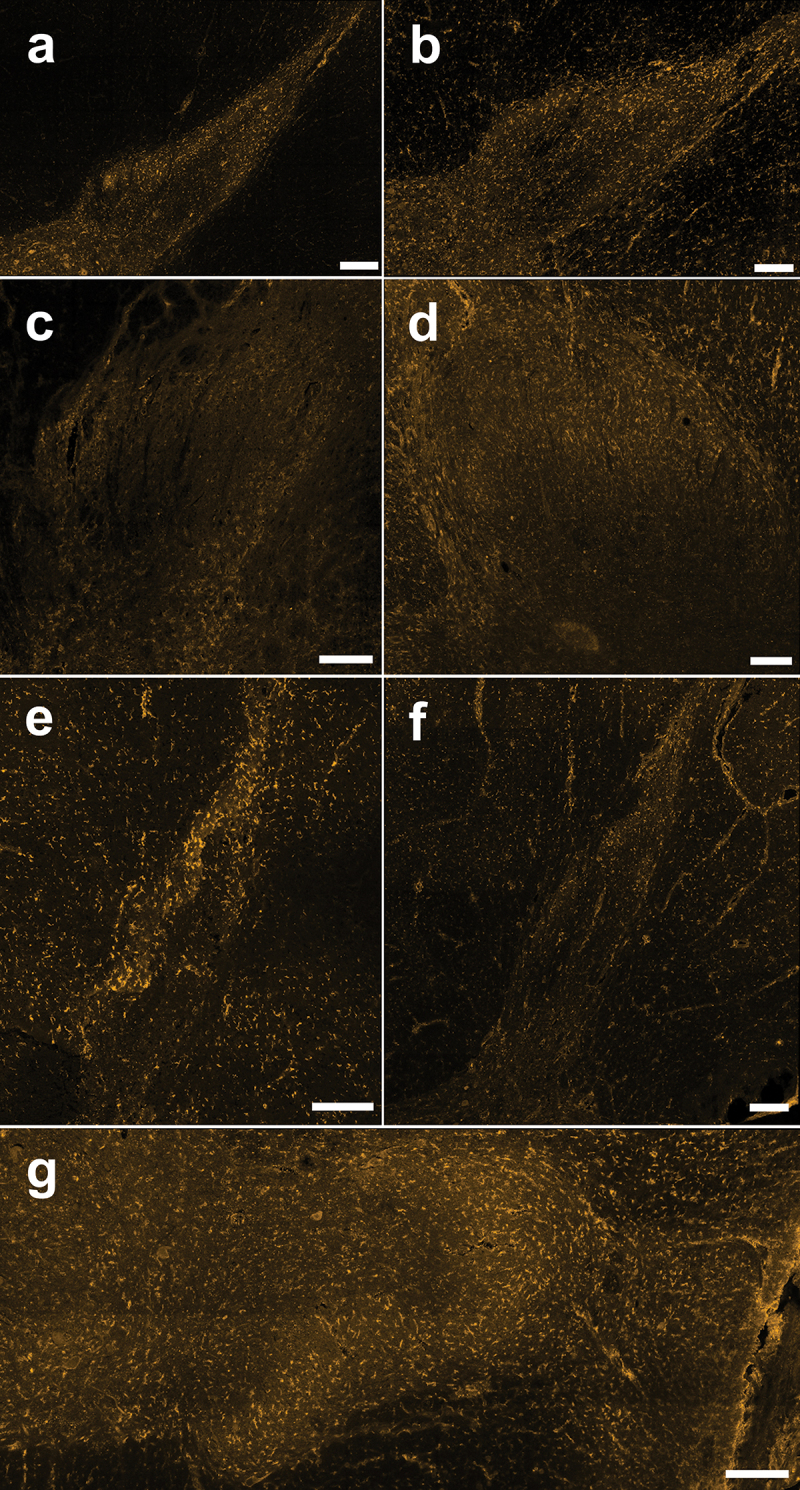
Immunoreactivity of the CB1 receptor in thoracic and lumbar spinal cord sections from male human organ donors. (A)–(G) Representative confocal images (20× objective) of CB1 immunostaining from each male human (*n* = 7) showing neuropil staining that is especially pronounced in the SDH and cellular staining across the DH. Scale bar = 200 µm.

To bridge the gap between these rodent preclinical findings and a better understanding and potential targeting of spinal pain mechanisms in humans, we next investigated CB1 distribution in viable spinal cord tissue from human organ donors.^23,32^ Using parallel collection, staining, and analysis condition as performed in rats (see Methods), we studied CB1 localization and distribution in the SDH and DDH of the thoracic and lumbar human spinal cord (Figure 1b). As observed in rats, we found that CB1 immunoreactivity was mainly observed as punctate neuropil labeling within the SDH and DDH, with some somatic staining throughout the dorsal horn in both female and male human donors (Figures 5 and 6). CB1 staining intensity was consistent across the SDH mediolateral axis.

**Figure 7.**
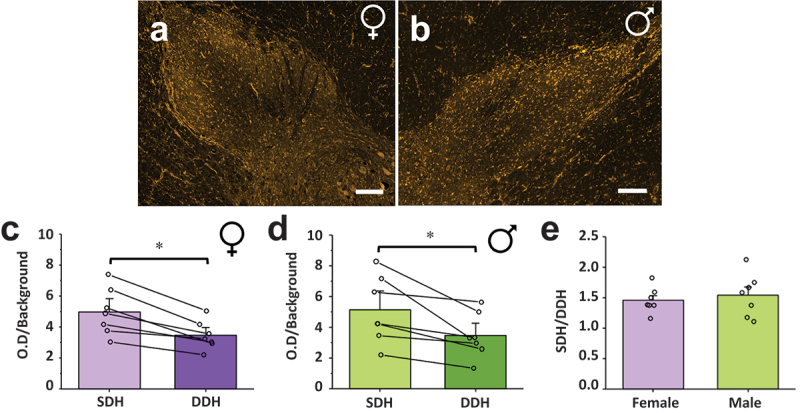
CB1 receptor immunoreactivity is increased in the SDH compared to the DDH in female and male humans. Representative confocal images of CB1 immunoreactivity in the dorsal horn of (A) female and (B) male human organ donors, side by side for direct comparison. The normalized CB1 optical density in the SDH of rats is statistically higher than the normalized optical density in the DDH for (C) female and (D) male humans. *P < .05. (E) A ratio comparing the normalized CB1 optical density of the SDH versus the DDH reveals that the increased optical density of CB1 receptors in the SDH of humans is conserved across sex. *n* = 6. Scale bar = 200 µm.

We next quantified the differential immunoreactivity of CB1 receptors in the SDH compared to the DDH of fixed human spinal cord sections. Spinal section staining and analyses were performed in duplicate for each donor, with average CB1 O.D. values and ratios taken for the SDH and DDH. As for rats, we found that CB1 immunoreactivity (O.D.) was significantly higher in the SDH compared to the DDH in both female (*n* = 7, *P* = .018, Figure 7,c) and male (*n* = 7, *P* = .018, Figure 7b,d) human donors. A two-way ART ANOVA^39,40^ comparing SDH values between sex and species revealed no significant differences between the factors or their interaction (sex *P* = .290; species *P* = .140; Sex * Species = 0.202; see Supplementary Table S1). A comparison of DDH values between sex and species revealed a significant difference between rat and human DDH levels (*P* = .043), whereas no significant difference was found between sex (*P* = .194) or the interaction of sex and species (*P* = .687; see Supplementary Table S1). To effectively compare CB1 dorsal horn distribution across sex, we created an SDH/DDH CB1 immunoreactivity ratio for both males and females (Figure 7e). The average SDH/DDH ratio was 1.45 ± 0.08 (*n* = 7) for females and 1.54 ± 0.13 (*n* = 7) for males, with no statistical difference between sexes (*P* = .61). Interestingly, the SDH/DDH ratio differs between species (sexes collapsed), with a significantly (*P* = .0077) higher ratio for rats compared to humans (Figures 4e, 7e, Supplementary Table S1). However, these subtle species differences for CB1 O.D. in the DDH and the SDH/DDH ratio may potentially be accounted for by changes in experimental and/or biological factors, such as differential levels of tissue myelination, changes in antibody binding efficiency, and/or distinct dorsal horn morphology between the two species. We therefore conclude that the preferential localization of CB1 receptors to the SDH is conserved from rats to humans, with no difference across sex for either species.

### CB1 Immunoreactivity Is Prominently Localized to Postsynaptic Dorsal Horn Neurons in Both Rats and Humans

**Figure 8.**
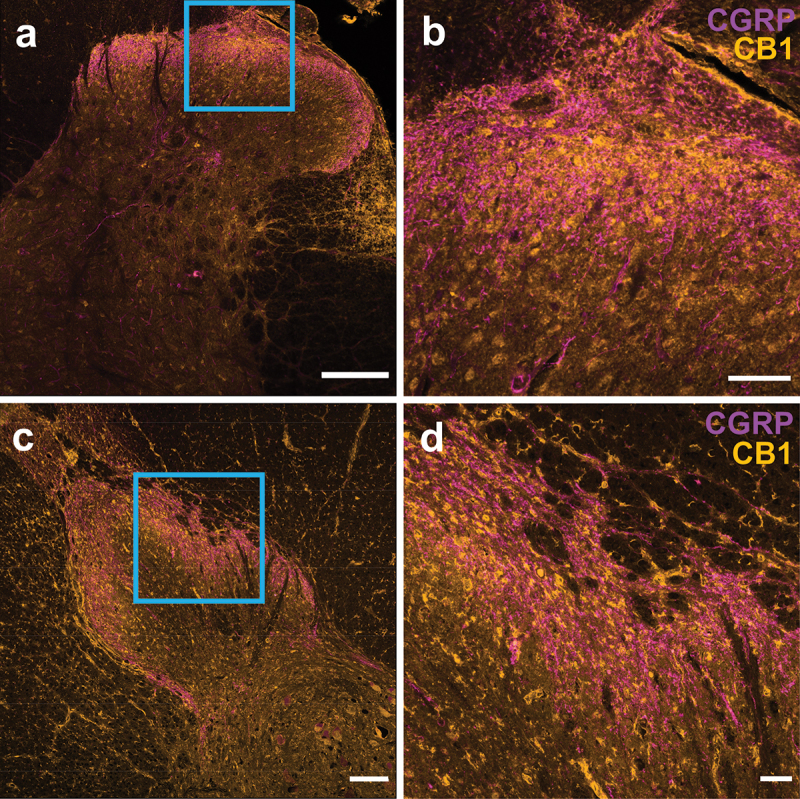
CB1 receptors are not primarily localized to peptidergic CGRP afferents. Overlay of CB1 receptor immunoreactivity (yellow) and CGRP immunoreactivity (purple) in (A), (B) rat and (C), (D) human spinal sections. A section of the SDH is shown at higher magnification (B), (D) to display localization patterns between CB1 and CGRP. Scale bar = 200 μm (A), (C). Scale bar = 50 μm (B), (D).

**Figure 9.**
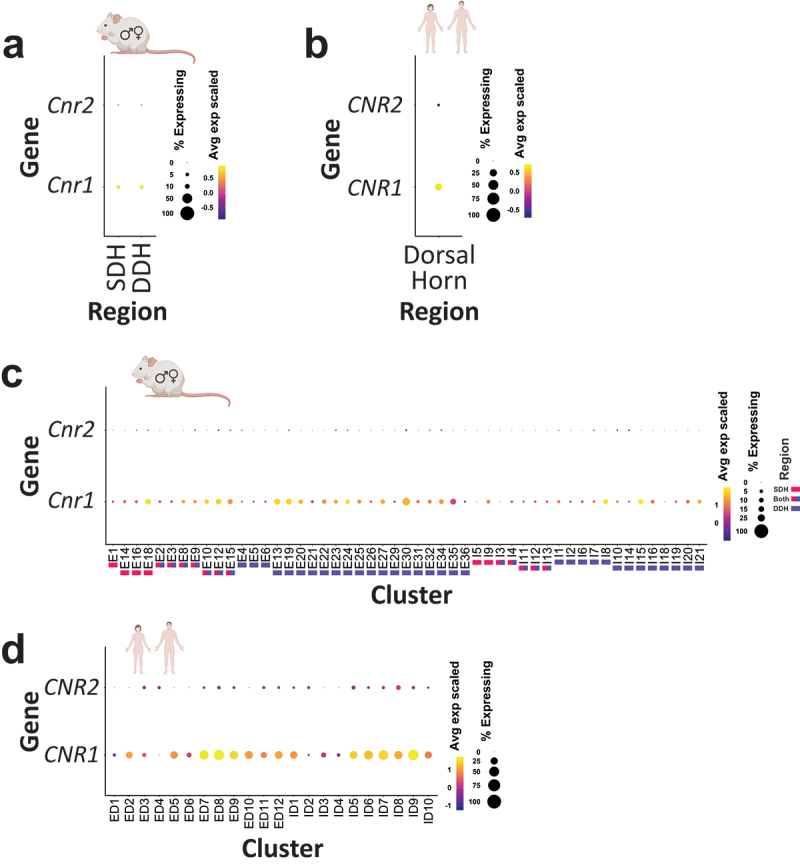
Single-cell/nucleus RNA sequencing of mouse and human spinal cord tissue reveals robust expression of the gene encoding CB1 (*CNR1*), but not CB2 (*CNR2*), in subsets of dorsal horn neurons. Dot plots showing the average expression and percent expressed for the genes encoding CB1 (*CNR1*) and CB2 (*CNR2*) in (A), (B) pooled and (C), (D) individual dorsal horn neuronal subpopulations from (A), (C) mouse^36^ and (B), (D) human^37^ single-cell/nucleus RNA sequencing data sets. The average expression is represented as the color of the dot and is a *z*-score scale of log_10_(CPM +1) counts. The percent expressed is represented by the size of each dot. Note the larger average expression of *Cnr1* (CB1 transcript) in SDH neurons compared to DDH neurons in mice. The colored blocks underneath the neuronal clusters indicate whether it was categorized to consist of cells from the SDH, DDH, or both based on information from Russ et al.^36^ Small illustrative mouse and human icons were created using BioRender.

In contrast to the canonical role of CB1 receptors being activated at presynaptic CNS terminals in retrograde mechanisms of plasticity,^20^ several rodent studies have reported that CB1 is highly expressed in second-order dorsal horn neurons, including within their somatic and dendritic compartments.^16,18,44^ This aligns with our observations of neuropil and somatic localization of CB1 immunoreactivity in the male and female rat and human dorsal horn (Figures 2, 3, 5 and 6). Indeed, when we qualitatively investigated the colocalization of CB1 receptors to CGRP-labeled peptidergic presynaptic afferents, we observed only a moderate degree of colocalization between CB1 and these nociceptive afferents in both rat (Figure 8a) and human (Figure 8b) spinal cord. To further validate this finding of prominent expression of CB1 receptors in postsynaptic dorsal horn neurons of rodents and humans, we analyzed recent single-cell/nuclei RNA sequencing data sets from mice^36^ and humans.^37^ We found that the gene encoding CB1 receptors, *CNR1*, but not the gene encoding CB2 receptors, *CNR2*, is highly expressed in dorsal horn neurons of mice (Figure 9a) and human organ donors^37^ (Figure 9b). In alignment with our finding of preferential localization of CB1 protein to the SDH in rodents and humans, we found higher average expression of *Cnr1* in SDH versus DDH mouse neurons (Figure 9a). Interestingly, in both mice (Figure 9c) and humans (Figure 9d), we found a large degree of heterogeneity of CB1-encoding gene expression between subtypes of excitatory and inhibitory dorsal horn neurons, with high percentage and average expression in some neuron clusters and minimal *CNR1* expression in other neuron subpopulations.

## Discussion

In this study, we characterized the expression of CB1 cannabinoid receptors in spinal pain processing regions of male and female rats and humans. To accomplish this, we adapted and optimized rodent CB1 immunohistochemical and confocal imaging protocols for successful immunostaining of highly viable spinal cord tissue from human organ donors.^32^ We found that CB1 was preferentially localized to the SDH within spinal cord slices from male and female rats and humans, with a pronounced expression of these potential pain-modulating receptors in postsynaptic dorsal horn neurons of both species.

A primary aim of this study was to examine the level of localization of CB1 receptors to the SDH in comparison to the DDH in rodent and human male and female spinal cord tissue. Previous rodent studies have shown that CB1 receptor expression is increased in the SDH compared to the DDH across all four levels of the spinal cord,^18,44^ so we used both thoracic and lumbar samples for our human tissue experiments. Consistent with previous findings, our results show increased CB1 receptor localization to the SDH compared to the DDH in rodent and human spinal cord samples. It should be noted that CB1 staining in this study was restricted to young adult rats (3–4 months old) but included a broader range for human spinal cords ranging from young adults into aged individuals (20–70 years old). Although we did not observe any change in CB1 dorsal horn localization across human adult ages (data not shown for privacy reasons), further experiments should investigate whether CB1 receptor distribution is conserved between young adult and aged rats. These studies could also use co-localization and cell count analysis to complement the more general CB1 O.D. quantification method used here. Studies examining cannabinoid receptor expression in the spinal cord have failed to compare across sex.^16,18,44,45^ In our study, we used equal numbers (*n* = 6) of males and females for both rodent and human staining experiments and found that the preferential localization of CB1 receptors to the SDH was conserved across sex in both species.

At a cellular level, we found that CB1 receptor immunoreactivity is preferentially observed as punctate labeling in the rat and human dorsal horn. This aligns with previous studies identifying predominant CB1 immunopositive puncta in the SDH of rat spinal sections.^16,18,44,45^ However, beyond punctate (i.e., neuropil) staining, we identified significant somatic CB1 staining throughout the rat and human dorsal horn. This observation has been found in several rodent studies,^16,18,44,45^ but the degree of localization of CB1 receptors to postsynaptic dorsal horn neurons remains variable and controversial. This could potentially be accounted for by the type of CB1-targeting antibody used. In our study, we used a CB1 knockout-validated polyclonal antibody, which has the affinity for multiple epitopes of the same antigen, compared to monoclonal antibodies that only have specificity for one epitope. To validate the finding of dorsal horn neuronal CB1 receptor expression using a distinct experimental approach, we analyzed single-cell/nuclear RNA sequencing data sets from both rodents and humans. This verified robust expression of CB1 receptor RNA (*CNR1*) across subsets of postsynaptic dorsal horn neurons in both mice and humans.

The finding of robust somatic CB1 staining suggests that CB1 receptors are located at both pre- and postsynaptic locations within spinal nociceptive circuits. To further investigate this, we examined co-localization between CB1 receptors and CGRP, a neuropeptide found on peptidergic primary afferents^46^ that is important for the modulation of spinal nociceptive processing.^47^ In agreement with several rodent studies,^18,44,48^ we qualitatively found that CB1 receptors do not co-localize with CGRP for the majority of rat SDH puncta. Importantly, we also show that this lack of enrichment of CB1 receptors at peptidergic nociceptive afferents is conserved from rats to humans.

It is also plausible that CB1 immunoreactivity within spinal nociceptive circuits is not limited to pre- and postsynaptic neuronal sites. Immunohistochemical studies in rodents have demonstrated robust colocalization of CB1 receptors within both astrocytes and microglia of the superficial dorsal horn.^49,50^ Approximately 50% of astrocytes and 80% of microglia are positively labeled with CB1 receptors.^16^ Astrocytes can produce 2-arachidonoylglycerol, which is a full agonist for the CB1 receptor.^51,52^ Immunohistochemical analyses demonstrated direct associations between astrocytes, CB1 receptors and diacylglycerol lipase-α, the precursor for 2-AG, suggesting that endocannabinoids, and particularly 2-AG play an important role in the communication between neurons and astrocytes.^49^ Further understanding the relationships between the cannabinoid system and glial cells will be essential in identifying potential therapeutic targets relating to physiological and pathological mechanisms of spinal pain processing.

The functional roles of CB1 presynaptic and postsynaptic receptors in modulating mechanisms of spinal nociception remain to be systematically investigated. Because CB1 receptor agonists decrease nociceptive DRG neuronal activity,^45,53–55^ it has been proposed that activation of these receptors will inhibit nociceptive afferents in the dorsal horn, resulting in dampened pain transmission.^56^ However, a recent study showed that analgesia produced by spinal administration of ∆^9^-THC was dependent on Cav3.2 T-type channels and not CB1 or CB2 receptors in male mouse models of inflammatory and neuropathic pain.^57^ Thus, whether cannabis components mediate analgesia partially through the activation of spinal CB1 receptors remains an open question.

Given that the majority of CB1 receptors in the SDH did not co-localize with CGRP, this suggests that CB1 may also be acting on nonpeptidergic neuron terminals and/or acting postsynaptically. Future immunohistochemistry experiments should target the colocalization of CB1 receptors with other peptidergic afferents such as neurokinin A and substance P, along with nonpeptidergic afferents such as the purinergic receptor P2X3 and isolectin B4 (for rodents only). Moreover, because our sequencing analyses reveal pronounced expression of CB1 within subsets of excitatory and inhibitory mouse and human dorsal horn neurons, future functional electrophysiological and/or imaging experiments should investigate the differential effects of CB1 receptor activation on subtypes of nociceptive dorsal horn neurons. In this context, researchers should use translationally relevant rodent *in* *vivo* models of chronic pain in combination with human spinal tissue models of pain pathology^23,32^ to investigate how CB1 (and potentially CB2) receptor expression and function are altered to drive neuronal hyperexcitability in spinal mechanisms of pathological pain.
